# Advancing Research to Eliminate Mental Illness Stigma: The Design and Evaluation of a Single-Arm Intervention Among University Students in Singapore

**DOI:** 10.3389/fpsyg.2020.01151

**Published:** 2020-06-03

**Authors:** Mythily Subramaniam, Shazana Shahwan, Edimansyah Abdin, Chong Min Janrius Goh, Wei Jie Ong, Gregory Tee Hng Tan, Nawira Baig, Ellaisha Samari, Kian Woon Kwok, Siow Ann Chong

**Affiliations:** ^1^Research Division, Institute of Mental Health, Singapore, Singapore; ^2^Lee Kong Chian School of Medicine, Singapore, Singapore; ^3^Nursing Department, Institute of Mental Health, Singapore, Singapore; ^4^School of Social Sciences, Nanyang Technological University, Singapore, Singapore

**Keywords:** stigma, social distance, education, contact, depression, knowledge

## Abstract

**Background:**

Anti-stigma interventions for school and college students have been studied in several countries, but to the best of our knowledge, this has not been addressed in Singapore. The current study was designed to address this lacuna and aimed to evaluate the effectiveness of an anti-stigma intervention focusing on depression among university students in Singapore.

**Methods:**

A one-off intervention comprising education and personal contact with a person with lived experience of depression was carried out in nine consecutive sessions over 6 months (October 2018 to April 2019) among 390 university students. Knowledge of depression and extent of stigma toward mental illness were assessed pre- and post-intervention as well as at 3-month follow-up.

**Results:**

The intervention was effective in improving depression knowledge (*d* = 1.09; *p* < 0.001), as well as reducing social distancing (*d* = 0.54; *p* < 0.001) and personal stigma (dangerous/undesirable: *d* = 0.60; *p* < 0.001 and weak not sick: *d* = 0.10; *p* < 0.033) pre- to post-intervention as well as pre- intervention to 3-month follow-up (*p* < 0.005).

**Limitations:**

While 3-month follow-up data indicates favorable medium-term impact on knowledge and stigma; the study lacks long-term follow-up to examine the impact of anti-stigma interventions across time. The data were collected through self-report measures; however, social desirability bias is possible despite the assurances of confidentiality.

**Conclusion:**

Given the impact of the intervention, there is a need to consider the feasibility, challenges, and enablers of implementation of such interventions into the curriculum of university students to ensure a broader and sustained outreach and stigma reduction.

## Introduction

The stigmatization of people with mental illness is a global and multidimensional phenomenon (Angermeyer and Dietrich, 2006; Thornicroft et al., 2009). Crocker et al. (1998) suggested that stigma occurs when a person possesses or is believed to possess “some attribute or characteristic that conveys a social identity that is devalued in a particular context” (p. 505). Corrigan and Shapiro (2010) defined stigma as a phenomenon comprising three constructs – (a) stereotypes (b) prejudices, and (c) discrimination. Stereotypes are oversimplified generalizations about people belonging to a specific group and are often culturally determined. These include preconceptions about the traits or abilities of people belonging to a particular group. Thus stereotypes are cognitive perceptions of how persons belonging to one group are “different” from persons belonging to another group. Stereotypes lead people to see groups as overly homogenous and therefore, they fail to see the individual’s characteristics. It also leads the person to focus on that information, which is in line with the stereotype and ignore the ones that do not conform with it. Prejudice is a negative attitude based on stereotypes toward members of a specific group. Allport (1954, p. 7) defined prejudice as “aversive or hostile attitude toward a person who belongs to a group, simply because he belongs to that group, and is therefore presumed to have the objectionable qualities ascribed to that group.” Stereotypes and prejudices lead to discrimination which is an unjustified, negative behavior toward members of a group. In the context of mental illnesses, stereotypes such as the belief that persons with mental illness are dangerous can lead to fear of people with mental illness (prejudice) which in turn can lead to them being turned down for jobs thereby limiting their access to employment (discrimination). Stigma thus has a tremendous impact on the well-being of people with mental illness and often results in poor access to healthcare (Mai et al., 2011; Gissler et al., 2013), exclusion from higher education (Lee et al., 2009), and unemployment (Sharac et al., 2010). Furthermore, the internalization of negative views resulting in self-stigma has been linked to low self-esteem and self–respect, as well as poorer quality of life, for people with mental illnesses (Świtaj et al., 2009; Ow and Lee, 2015; Corrigan et al., 2016; Picco et al., 2016).

Research in Singapore has identified significant public stigma toward those with mental illness (Yuan et al., 2016; Pang et al., 2017; Subramaniam et al., 2017). A study on Singaporean youth identified several misconceptions toward mental illnesses with a significant proportion stating that they would not want others to know about their mental illness should they have one, and felt that they would be stigmatized by their peers if they knew about their illness (Pang et al., 2017). Collectivist values, as well as the perceived “loss of face” if a person/family member is diagnosed with mental illness and the resultant loss of social capital, are concepts that are unique to Asian cultures (Yang and Kleinman, 2008; Papadopoulos et al., 2013). Entwined with these cultural factors is the importance given to the concept of meritocracy in Singapore. Young people may not want to be seen as belonging to the “Other” negatively stereotyped group and thus may avoid getting a diagnosis and accessing treatment. Those pursuing their education may also be fearful of the loss of social capital if their diagnosis is disclosed as discrimination may affect their future academic or employment prospects.

This is reflected in the fact that although mental disorders are prevalent in Singapore’s population (13.9%) especially among those aged 18–34 years (21.6%) (Subramaniam et al., 2020), and youth in Singapore identified mental health issues such as depression and anxiety as one of the top issues they faced (National Youth Council, 2019), there was a large treatment gap across all age groups (Subramaniam et al., 2019). Reasons for not seeking help included “not knowing where to seek treatment” as well as “concerns about what others would think” if they found out that the person was seeking treatment. Thus, suggesting that the treatment gap is influenced by both a lack of mental health literacy and stigma toward mental illness.

Several anti-stigma interventions have been developed to mitigate the negative impact of stigma across various populations such as the general public, police officers, healthcare professionals, and students (Gronholm et al., 2017). This current study aimed to design and evaluate the effectiveness of an anti-stigma intervention focusing on depression among university students in Singapore. Depression was chosen as it had the highest prevalence among the mental disorders examined in Singapore, and despite being well-recognised, it is associated with a significant treatment gap (Chong et al., 2016; Subramaniam et al., 2019, 2020). University students were chosen as the target group for the intervention after considering several factors. As stated earlier, mental illnesses, including depression, are higher in this population. Experiencing a mental illness in college has been associated with academic disengagement (Eisenberg et al., 2009) as well as suboptimal economic and social outcomes in later life (Goodman et al., 2011; Kawakami et al., 2012). Further, beyond encouraging the young person with a mental illness to seek help, interventions in this group have the potential to enlist peers who can encourage a distressed friend to seek help. Additionally, university or college students may become future leaders of communities with the influence and power to reduce stigma.

The effectiveness of the intervention was evaluated in terms of improving the knowledge of depression, and reducing stigma and social distance. We hypothesized that the intervention would result in improved knowledge, and reduce stigma and social distance toward depression and that these effects would persist at 3-month follow-up.

## Materials and Methods

### Sample

Students from a local university were invited to participate in the study. An e-mail was sent out by the university staff, asking students to indicate their willingness for participation in the study on a designated webpage through an Internet link. Additionally, the study was also advertised by putting up a post on the university’s Facebook groups with the Internet link, and by putting up posters with a QR code leading to the same designated webpage for those interested in participating in the study. As per the requirements of our ethics committee in all the outreach materials, we described the intervention as: “a study carried out to evaluate the effectiveness of an anti-stigma talk that focuses on mental illness, among university students in Singapore.” To keep the sessions interactive and depending on the size of the available room, participation was restricted to a maximum of 50–70 students. Those who indicated their willingness were sent consent forms by e-mail, and for students who were younger than 21 years, parental consent was also sought. On the day of the session, consent for each participant was taken by a research staff, and they were asked to clarify their doubts related to the study or study procedures. The study was approved by the institutional ethics committee (National Healthcare Group, Domain Specific Review Board).

### Intervention

Education, contact, and protest have been suggested as the core elements for reducing stigma according to the stigma reduction theory by Corrigan and Penn (1999). Education provides factual information about mental illnesses, replacing myths and stereotypes that individuals may harbor. Contact includes interactions with people who have a mental illness, which may challenge prejudices. Protest, where one identifies, highlights, and speaks out against prejudices and discriminatory acts toward those with mental illness, can also potentially reduce stigma (Corrigan et al., 2001). A systematic review by Yamaguchi et al. (2013) indicated that live or video-based contact with people with mental health problems were the most effective interventions in improving attitudes and reducing the desire for social distance. However, results from another review suggested that providing treatment information might enhance students’ attitudes toward the use of services (Gulliver et al., 2012). A systematic review by Thornicroft et al. (2016) found that interventions primarily involving either mental health education or education combined with contact with someone who has a mental health problem resulted in an improvement in knowledge and attitudes over the short-term, though this effect diminished with time.

The intervention for the current study was designed based on the stigma reduction theory and findings that interventions involving education combined with direct contact are effective in reducing stigma. The one-off intervention comprised a single session. The educational component comprised a lecture on the prevalence, symptoms, and biopsychosocial causes of depression. Factual information on treatment options as well as avenues for help-seeking was also provided. The contact component of the intervention comprised a sharing session by a person with lived experience of mental illness about the clinical aspects of her depression, her challenges in accepting her illness and in seeking help, and concluded with a sharing of her recovery journey. The person had worked as a youth ambassador for a mental health service provider in Singapore and was trained to educate people about mental health issues (Community Health Assessment Team, 2014). This was followed by a Question and Answer (Q&A) session with a consultant psychiatrist, a mental health research expert and the person with lived experience where students could clarify their doubts or ask for more detailed information related to the presentations.

In order to meet the target sample size, nine sessions were held over a period of 6 months (October 2018 to April 2019). All the sessions were held in the evenings after classes to facilitate participation. The interval between sessions was usually one week but longer breaks were scheduled during the exam period and term break. Consistency was maintained across all the sessions by using the same material, which was delivered as a powerpoint presentation usually by the same person (all except three sessions), sharing by the same person with lived experience of mental illness, and the same members of the research group participating in the (Q&A) sessions. Each session lasted for about an hour.

### Questionnaires

Data was collected through a series of paper-and-pen questionnaires. These questionnaires were administered before and after the intervention. The students were asked to provide socio-demographic information, and this was followed by a short vignette that students were instructed to read before answering the other questionnaires. The vignette described a person (named Adam) with depression, and it has been used previously in a population-wide study in Singapore (Chong et al., 2016). The questionnaires used were as follows:

(i) Depression literacy questionnaire (Griffiths et al., 2004): This measure consists of 22 items, which includes statements assessing the respondents’ knowledge about depression. For each statement, respondents will select what they believe to be the correct response from three possible choices (true, false, or I do not know). Respondents score 1 point for each correct answer, and total scores ranged from 0 to 22 with higher scores indicating higher literacy for depression. For the rest of this article, we have referred to depression literacy scores as knowledge scores.
(ii) The Personal Stigma subscale of the Depression Stigma Scale (DSS) (Griffiths et al., 2004): This scale measures the respondents’ attitudes toward depression by asking them to indicate how strongly they personally agree with nine statements about depression. For the purposes of this study, only the eight-item DSS-personal subscale was used (“I would not vote for a politician if I knew they had a mental illness” item was not included). Responses to each item are measured on a 5-point scale (ranging from 1 “strongly disagree” to 5 “strongly agree”). This questionnaire has been validated in the local population and shown to comprise two distinct dimensions comprising “weak-not-sick” and “dangerous/unpredictable” (Subramaniam et al., 2017).
(iii) The Social Distance scale (Link et al., 1999): This scale measures the self-reported willingness to make social contact with the person described in the vignette. Responses to each item were measured on a 4-point scale (ranging from 1 “definitely willing” to 4 “definitely unwilling.” The scale score is calculated by summing item scores, where higher scores indicate a greater desire for social distance.

Sociodemographic information about the respondent, namely, age, sex, nationality, ethnicity, and religion, was collected. Data related to participants’ university experiences were also collected such as year and discipline of study. As some students may be/have been involved in volunteer groups or campus peer-helping activities, a question on such involvements was also included. They were also asked if they had any close friends/family members who had been diagnosed with a mental illness.

Students were asked to provide an e-mail address at which they could be contacted for the 3-month follow-up. The set of questions that were used post-intervention were sent to the participants at the 3-month follow-up. A mass reminder email was sent a week following the 3-month follow-up email.

### Sample Size Estimation

The estimation of sample size in this study was performed using the two means formula for paired data with power and alpha of 80 and 5%, respectively. The sample size was calculated with reference to another study (Ahuja et al., 2017), where 50 college students were recruited for a one-time educational and contact-based intervention, and changes in their attitudes toward mental illness were tracked by comparison of their CAMI scores pre-and post-intervention. Based on the means and standard deviations of the 4 CAMI subscales: authoritarianism, social restrictiveness, benevolence, and community mental health ideology at the pre- and post-intervention of the aforementioned study, we arrived at an estimated sample required size for our study. We found that we would need at least 233 subjects to be able to reject the null hypothesis that the means of the CAMI subscales are equal between the pre- and post-intervention assessments. After considering approximately 40% loss to follow-up (40/100 × 233 = 93), a final sample size of 326 (93 + 233 = 326) was determined to be sufficient for the study.

### Statistical Analysis

All analyses were conducted with SAS software version 9.4. Means and standard deviations were calculated for continuous variables, while frequencies and percentages were calculated for categorical variables. Linear mixed models were used to assess the effects of the intervention and to account for missing data, individual heterogeneity and repeated measurements on the same individuals over time. The “time” variable was included in the linear mixed models as both random and fixed effects to adjust for the overall and the individual variations in the stigma scores throughout time. Linear and quadratic effects were tested as both random and fixed parameters, along with interaction terms with other covariates. The models were first done unconditionally (i.e., without covariates) to compare pre-intervention, post-intervention, and 3-month follow-up scores on the personal stigma (weak-not-sick and dangerous/undesirable), and social distance scale. This was followed by using baseline socio-demographic factors such as age, gender, ethnicity, year of study, whether they have close friends or family member who had a mental illness and having experience in the past in the mental health field (e.g., involvement in volunteer groups or campus peer-helping activities) as time-invarying covariates as well as changes in knowledge of depression over time as a time-varying covariate. The effect of any potential factors that might influence the rate of change in the scores over time was explored using interaction terms between time and each covariate. Means and standard deviations for personal stigma and social distance scores at different time assessments were also calculated. Effect sizes were calculated comparing pre-intervention scores to the post-intervention and 3-month follow-up scores using Cohen’s *d* = (Mean time 2−Mean time 1)/Pooled SD. It has been suggested that a value of *d* of 0.2–0.5 represents a small effect, a value of 0.5–0.8 represents a medium/moderate effect, and a value of *d* of 0.8 or higher represents a large effect. Statistical significance for all analyses was set at the conventional alpha level of *p* < 0.05, using two-tailed tests.

## Results

The characteristics of the sample are presented in Table 1. The pre-intervention sample consisted of 390 students from a university in Singapore aged 18–31 years. The majority were females (60.3%), of Chinese ethnicity (82.8%), and 22.2% had past experience in the mental health field. 326 students completed the 3-month follow up assessments (retention rate = 83.6%).

**TABLE 1:** Baseline characteristics of the sample (*n* = 390).

	**Mean**	**SD**
**Age**		
(Range: 18–31)	22.3	2.3
**Gender**	**n**	**%**
Female	235	60.3
Male	155	39.7
**Ethnicity**		
Chinese	323	82.8
Malay	12	3.1
Indian	37	9.5
Others	18	4.6
**Year of study**		
Year 1	130	33.3
Year 2	97	24.9
Year 3	79	20.3
Year 4	84	21.5
**Course of study**		
STEM	228	58.5
Non-STEM	160	41.2
**Close friends or family member who has a mental illness**		
Yes	166	42.6
No	224	57.4
**Past experience within the mental health field**		
Yes	86	22.2
No	301	77.8

*STEM: science, technology, engineering, and math.*

### Depression Literacy

**FIGURE 1:**
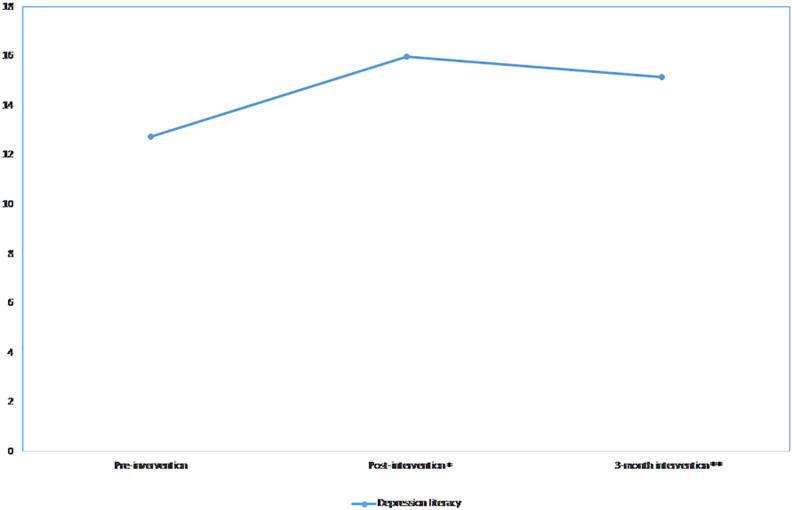
Mean depression literacy scores over time. *p < 0.05; **p < 0.01.

The linear (β = 9.5, *p*-value < 0.001) and quadratic (β = −2.1, *p*-value < 0.001) effects were significant, indicating that the knowledge scores increased post-intervention and slightly reduced over the 3-month follow-up period (Figure 1). A significant difference was observed in knowledge scores when comparing the pre-intervention to the post-intervention scores (*p*-value < 0.001) and the 3-month follow-up scores (*p*-value < 0.001). The effect size was reduced from *d* = 1.09 at post-intervention to *d* = 0.75 at the 3-month follow-up compared to post-intervention (Table 2). The significant linear and quadratic effects remained the same after adjusting for all covariates (Table 3). When interaction terms were added in the model, significant interactions were found between time and those who had family or friends with mental illness. Those who had family or friends with mental illness tended to have a lesser increase in the knowledge scores at the post-intervention (β = −3.3, *p*-value = 0.026) and a lesser decrease in the knowledge scores (β = 0.8, *p*-value = 0.025) at the 3-month follow-up.

**TABLE 2:** Personal stigma and social distance scores at pre-intervention, post-intervention, and 3-month follow-up.

	**Pre-intervention**		**Post-intervention**		**Effect size^b^**	***p*-Value^b^**	**3-month follow-up**		**Effect size^c^**	***p*-Value^c^**	**Effect size^d^**	***p*-Value^d^**
			
	**Mean**	**SD**	**Mean**	**SD**	**Cohen’s *d*^a^**		**Mean**	**SD**	**Cohen’s *d*^a^**		**Cohen’s *d*^a^**	
Weak-not-sick	4.5	2.3	4.2	2.1	0.10	0.033	4.0	2.4	0.09	0.096	0.18	0.003
Dangerous/Undesirable	5.7	2.7	4.1	2.7	0.60	<0.001	4.6	2.6	0.17	0.009	0.43	<0.001
Social distance	10.9	2.9	9.4	2.7	0.54	<0.001	9.8	2.8	0.12	0.042	0.41	<0.001
Depression knowledge	12.8	3.2	16.0	2.7	1.09	<0.001	15.2	3.2	0.75	0.875	0.28	<0.001

*^*a*^Cohen’s *d* = (Mean time 2−Mean time 1)/Pooled SD; unconditional linear mixed model = ^*b*^pre- vs. post-intervention, ^*c*^post vs. 3-month intervention, ^*d*^pre- vs. 3-month intervention.*

**TABLE 3:** Effects of intervention on personal stigma, and social distance.

	**Weak-not-sick**			**Dangerous/unpredictable**			**Social distance**			**Depression knowledge**		
				
	β	**95% CI**	***p*-Value**	**β**	**95% CI**	***p*-Value**	**β**	**95% CI**	***p*-Value**	**β**	**95% CI**	***p*-Value**
Intercept	5.1	(2.9-7.3)	<0.001	12.3	(9.7-14.9)	<0.001	17.7	(15.5-20.0)	<0.001	3.1	(0.4-5.8)	0.022
Time	1.1	(0.3-1.9)	0.008	–1.9	(−3.4 to −0.4)	0.013	–2.9	(−4.3 to −1.5)	<0.001	10.8	(8.9-12.7)	<0.001
Time^2^	–0.3	(−0.5 to −0.1)	0.005	0.7	(0.4-1.0)	<0.001	0.6	(0.2-0.9)	<0.001	–2.4	(−2.9 to −1.9)	<0.001
Depression knowledge	–0.1	(−0.2 to −0.1)	<0.001	–0.04	(−0.2 to 0.1)	0.484	–0.2	(−0.2 to -0.1)	<0.001			
Age (years)	0.05	(−0.1 to 0.2)	0.361	–0.1	(−0.2 to -0.1)	0.002	–0.1	(−0.1 to 0.03)	0.198	0.02	(−0.1 to 0.1)	0.738
Female vs. Male	–0.1	(−0.5 to 0.3)	0.746	–1.1	(−1.4 to -0.7)	<0.001	–0.5	(−0.9 to -0.1)	<0.008	0.3	(−0.1 to 0.7)	0.161
Malay vs. Chinese	–1.2	(−2.1 to -0.1)	0.022	–0.8	(−1.8 to 0.1)	0.091	–1.2	(−2.1 to -0.3)	0.012	–1.0	(−2.0 to 0.04)	0.060
Indian vs. Chinese	–0.9	(−1.2 to −0.5)	0.005	–0.5	(−1.0 to 0.05)	0.076	–1.0	(−1.5 to -0.5)	<0.001	–1.1	(−1.7 to −0.5)	<0.001
Others vs. Chinese	0.4	(−0.4 to 1.3)	0.347	0.7	(−0.1 to 1.5)	0.075	0.8	(−1.6 to 0.01)	0.054	–0.8	(−1.6 to 0.1)	0.091
Year of study (years)	–0.1	(−0.3 to 0.1)	0.118	0.2	(0.03-0.3)	0.018	0.1	(−0.04 to 0.3)	0.13	0.02	(−0.2 to 0.2)	0.862
Family or friends with mental illness (Yes vs. No)	–0.9	(−1.2 to -0.5)	<0.001	–0.6	(−0.9 to -0.3)	<0.001	–2.2	(−3.0 to -1.3)	<0.001	3.6	(1.0-6.2)	0.006
Past experience in mental health field (yes vs. no)	–0.5	(−0.9 to -0.04)	0.034	–0.7	(−1.1 to -0.3)	<0.001	–0.8	(−1.2 to −0.4)	<0.001	1.6	(1.2-2.0)	<0.001
**Interaction terms**												
Time × Depression knowledge				–0.1	(−0.1 to −0.03)	0.006						
Time^2^ × Family or friends with mental illness										0.8	(0.1-1.6)	0.025
Time × Family or friends with mental illness							0.6	(0.2-1.0)	0.005	–3.3	(−6.3 to −0.4)	0.026

### Weak-Not-Sick Scores Over Time

**FIGURE 2:**
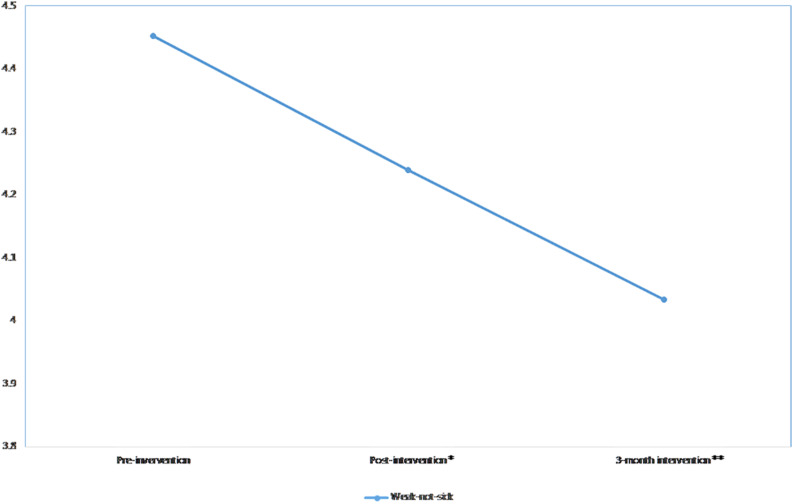
Mean weak-not-sick scores across time. *p < 0.05; **p < 0.01.

There was a significant downward linear trend over time in the weak-not-sick scores (β = −0.2, *p*-value = 0.003), indicating that the scores decreased post-intervention and remained low during the 3-month follow-up period (Figure 2). A significant difference was observed in weak-not-sick scores when comparing the pre-intervention scores to the post-intervention (*p*-value = 0.033) and the 3-month follow-up scores (*p*-value = 0.003). The effect size was slightly reduced from *d* = 0.10 at post-intervention to *d* = 0.09 and after 3 months. After the addition of covariates in the linear mixed model, significant linear and quadratic effects were observed in the data where the scores significantly increased post-intervention (β = 1.1, *p*-value = 0.008) and decreased at 3-month follow-up (β = −0.3, *p*-value = 0.005) (Table 3). When interaction terms between time and covariates were added, no interaction terms were significant.

### Dangerous/Unpredictable Scores Over Time

**FIGURE 3:**
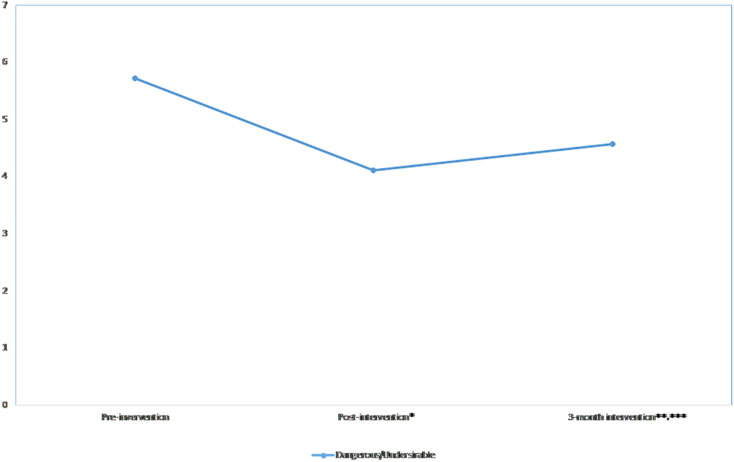
Mean dangerous/predictable scores across time. *p < 0.05; **p < 0.01, ***p < 0.001.

The linear (β = −4.6, *p*-value < 0.001) and quadratic (β = 1.0, *p*-value < 0.001) effects were significant, indicating that the dangerous/unpredictable scores decreased post-intervention and increased over the 3-month follow-up period (Figure 3). The scores significantly decreased at post-intervention (*p*-value < 0.001) and the 3-month follow-up (*p*-value < 0.001) when compared to the pre-intervention scores, and significantly increased when the 3-month scores were compared to the post-intervention scores (*p*-value = 0.009). The effect size was moderate (*d* = 0.60) at the post-intervention and small at the 3-month follow-up (*d* = 0.17) (Table 2). The significant linear and quadratic effects remained the same after adjusting for all covariates (Table 3). When interaction terms were added in the model, significant interactions were found between time and knowledge scores; increased knowledge scores tended to further decrease the dangerous/unpredictable scores (β = −0.1, *p*-value = 0.006) at post-intervention. However, no significant interactions were found at 3-month follow-up.

### Social Distance

**FIGURE 4:**
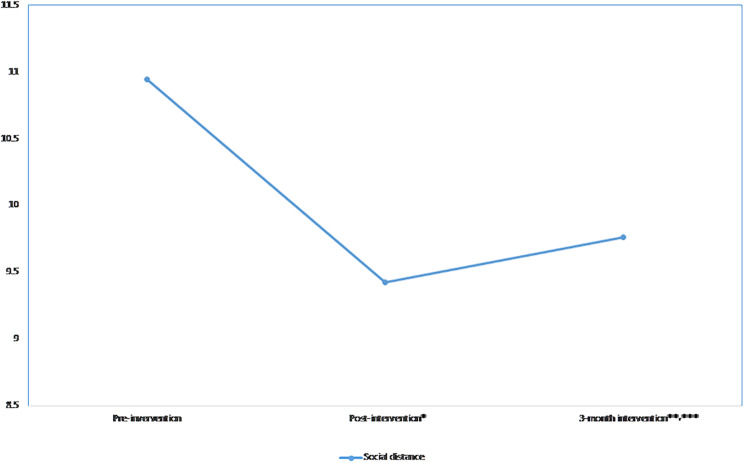
Mean social distance scores across time.

The linear (β = −4.2, *p*-value < 0.001) and quadratic (β = 0.89, *p*-value < 0.001) effects were significant, indicating that the scores decreased post-intervention and increased at the 3-month follow-up (Figure 4). The scores significantly decreased at post-intervention compared to pre-intervention and significantly increased at the 3-month follow-up when compared to the post-intervention scores (*p*-value = 0. 038). The effect size was medium (*d* = 0.54) at post-intervention and small at 3-month follow-up (*d* = 0.12). The significant linear and quadratic effects remained after adjusting for all covariates. A significant interaction was observed between time and those who had family or friends with mental illness (Table 3). Those who had family or friends with mental illness (β = 0.6, *p*-value = 0.005) tended to have a lesser decrease in social distance scores at post-intervention than their counterparts.

## Discussion

This research focusing on university students examined the effects of an educational and contact-based intervention on personal stigma and social distancing toward depression immediately following the intervention and 3 months after the intervention. The findings supported our hypotheses. There was a significant reduction in personal stigma and social distancing from pre- to post-intervention, as well as from pre-intervention to the 3-month follow-up. Effect sizes were medium for the dangerous/unpredictable dimension as well as social distance and considered trivial for the weak-not-sick dimension. Our findings are different from that of Kosyluk et al. (2016), who found small effect sizes of stigma change at post-intervention among university students. However, the authors compared educational interventions with social contact, and the combined approach adopted by this study could have resulted in a better outcome. Few studies have examined and reported stigma change in the follow-up period. Campbell et al. (2011) examined the impact of a short psychosocial intervention that involved mental health education and contact with an ex-service user to reduce discrimination toward psychosis in 43 pupils and found that the gains in the discrimination reduction were not sustained at 10-week follow-up. A review by Mehta et al. (2015) concluded that the effectiveness of anti-stigma interventions after 4 weeks was modest in terms of increasing knowledge and reducing stigmatizing attitudes, and emphasized the need for robust studies that examine long-term outcomes as well as to explore the use of booster interventions for sustainability.

The mean scores in the sample for the weak-not-sick and dangerous/unpredictable dimensions at baseline were 4.5 and 5.7, respectively. These significantly contrasted with the mean scores observed in a nationwide study on mental health literacy, where the adult sample endorsed more stigmatizing attitudes toward those with depression in the two domains of DSS with mean scores of 10.6 and 10.8, respectively (Subramaniam et al., 2017). The lower pre-intervention scores in the student sample in the domains of the DSS, indicating lower stigmatizing attitudes are encouraging, and they may also explain the small-medium effect size over time. Interestingly, and somewhat surprisingly, social distance scores were not different in the population and student samples, with both samples having a mean score of 10.9 (pre-intervention scores for the university group). Social distancing may be influenced by many factors (Lauber et al., 2004), including perceptions of dangerousness and causal beliefs. Although the students had lower scores on the dangerous/undesirable domain, this did not reduce social distancing, suggesting that social distancing may be more ingrained, and there could be cultural aspects associated with it. A study by Cleveland et al. (2013) found that while psychosocial causal attributions were associated with lower social distancing, the attribution to personal factors such as lack of discipline, weakness of character, and wrong lifestyle were associated with higher social distancing. Studies have found that Asians tend to endorse personal factors as a cause for mental illness (Nakane et al., 2005; Pang et al., 2018). A previous population-wide study in Singapore found that “Personality” (being a nervous person and having a weak character) was perceived to be a significant causal attribute, with 89.1% of the population attributing it as a cause of depression (Pang et al., 2018). While this study did not examine the role of causal attributes in social distancing, these beliefs may play a role. On the other hand, Norman et al. (2008) suggested that social distancing is associated with perceived normative expectations about the behavior. Thus, if it was perceived that the illness would result in embarrassing behavior that would not be favorably looked upon by those considered important to the respondent, or that they would not engage with this person, greater would be the social distancing endorsed by the respondent. The respondents may have also felt that associating with a person with mental illness may result in “courtesy stigma,” which is defined as the distancing and rejection faced by individuals who are associated with members of a socially devalued category (Goffman, 1963). Young people may be even more sensitive to being “othered” by their peers and thus may not be willing to associate with a person with mental illness. However, it was encouraging that the intervention resulted in a reduction in social distancing even 3 months post-intervention though the effect size was small.

There was a significant increase with a large effect size in the depression knowledge from pre- to post-intervention, which, however, declined after 3 months. The educational intervention provided factual information and may have served to challenge existing stereotypes about depression (Corrigan and Shapiro, 2010; Economou et al., 2012). Depression knowledge at baseline was associated with lower stigmatizing attitudes; there was also a significant time × knowledge interaction at post-intervention where increased knowledge scores were associated with lower dangerous/unpredictable scores. However, we were not able to detect any significant interaction at the 3-month point, which may have been due to the decrease in the knowledge scores from post-intervention to 3-month follow-up.

Our results also highlight the importance of contact in lowering stigma. Reinke et al. (2004) suggested that contact is often most helpful when the contact person is an individual of a similar age to the participants and only “moderately” disconfirming of stereotypes. In our study, the person with the lived experience while slightly older than the students was able to connect with the audience as she shared her experiences candidly; her narrative of struggles and successes resonated with them. Since the person had prior experience of sharing her story, she came across as someone who was confident and capable and answered the questions posed by the participants competently. Strategising the message of the educational intervention is equally important. Lam et al. (2005) in their interventional study to evaluate the impact of causal labels on mental illnesses found that individuals who were provided a psychological cause for the disorder rated patients as significantly more likely to be curable and significantly less likely to harm themselves. Concerning the current study, the lecture provided information on all probable causes; however, the emphasis was more on help-seeking, helping a peer in distress, various forms of treatment that are easily available in Singapore, and recovery.

While several covariates were significant across different measures of stigmatizing attitudes, the influence of two variables is noteworthy. While female gender was a significant covariate for stigmatising attitudes, we did not find a significant effect of being female on the response to the intervention. Similar results were found by Andrés-Rodríguez et al. (2017) who evaluated the “What’s Up” intervention in Catalan high schools, where female gender was associated with lower stigmatizing attitudes, but it did not influence the effect of the intervention. Another covariate that appeared to be significantly associated with lower stigma and higher knowledge scores was – having a close family or friend with a mental illness. It may be the case that having a close relationship with someone with a mental illness makes it easier for youths to feel empathy with them. They may, therefore, have lower levels of stigma than those who have no friends or close relatives with depression, as it has been observed that empathy is a key individual factor influencing attitude change toward mental illnesses (Couture and Penn, 2003). It is also possible that these youths have a better understanding of mental illnesses and have a more realistic understanding of someone with mental illness. However, these youths showed significantly lower improvement in social distancing and knowledge in response to the intervention. Andrés-Rodríguez et al. (2017) on the other hand, did not find any effect of the intervention on this group, although stigma levels were lower in youths who had friends or close relatives with the problem at baseline. The contact with the person with lived experience of mental illness may have been an eye-opener for those who did not have friends or family members with a mental illness leading to a significant attitude shift. This would have been much more than for someone who was already aware of the strengths and capabilities of a person with a mental illness.

A limitation of the study is that the present sample may not be representative of the overall population of university students in Singapore. Since this was a research study and participation was voluntary with the sessions conducted in the evening, there is a possibility that those who volunteered for participation may be more interested in learning about mental illnesses or be more empathetic toward those with mental illnesses. It is important to conduct similar studies across samples that are not self-selected such as by incorporating the intervention as part of a module in the curriculum so as to arrive at a more accurate understanding of the wider student population. While a 3-month follow-up is medium-term and informs us that there are positive gains from pre-intervention levels, long-term follow-up research will be important to examine whether the impact of anti-stigma interventions is maintained across time. The data were collected through self-report measures. It is possible that some participants provided socially desirable answers despite assurances of confidentiality. Lastly, while the intervention was successful in reducing negative attitudes and expressed social distance toward people with depression, it is uncertain if this change will translate into more positive behaviors.

## Conclusion

In conclusion, this research represents an important first step in the development and evaluation of a combined educational and contact-based intervention for reducing stigma toward depression. The findings suggest that the intervention results in both short- and medium-term benefits in terms of attitude change, although the stability of these benefits in the long term and their relation to behavior change are unknown. Corrigan (2011) has suggested that mental illness stigma reduction is most likely to be effective when it is targeted toward specific populations, is locally based and delivered, continuous, credible, and involves contact with people who have successfully managed their mental illness. Our intervention was planned carefully taking all these points into consideration. However, it was a one-off interaction and not sustained over a longer period of time. Further studies are needed to evaluate the long-term effectiveness of these interventions. The acceptability and effectiveness of web-based interventions need to be evaluated as these are less resource intensive. We also need to consider the feasibility, challenges, and enablers of implementation of such interventions into the curriculum of university students to ensure a wider and sustained outreach and stigma reduction.

## Data Availability Statement

The datasets generated for this study are available on request to the corresponding author.

## Ethics Statement

The study was reviewed and approved by National Healthcare Group Domain Specific Review Board, Singapore. Written informed consent to participate in this study was provided by the participants.

## Author Contributions

MS wrote the first draft of the manuscript. MS, EA, SC, and KW designed the study protocol. EA conducted the statistical analysis. ES and SS conducted the literature review. SS, CG, WO, and GT developed the educational intervention and provided inputs into the scales included in the study. NB planned and delivered the narrative of lived experience. All authors gave their intellectual input to the manuscript and have read and approved the final draft of the manuscript.

## Conflict of Interest

The authors declare that the research was conducted in the absence of any commercial or financial relationships that could be construed as a potential conflict of interest.
